# How do primary care providers and autistic adults want to improve their primary care? A Delphi-study

**DOI:** 10.1177/13623613231172865

**Published:** 2023-05-16

**Authors:** Eva B Warreman, Wietske A Ester, Hilde M Geurts, Robert RJM Vermeiren, Laura A Nooteboom

**Affiliations:** 1 Leiden University Medical Center Curium, the Netherlands; 2 Parnassia Psychiatric Institute, the Netherlands; 3 Sarr Autisme Rotterdam, the Netherlands; 4 University of Amsterdam, the Netherlands; 5 Dr. Leo Kannerhuis, Youz, Parnassia Psychiatric Institute, the Netherlands

**Keywords:** autism, barriers, general practice, healthcare access, primary care, recommendations

## Abstract

Autistic adults often experience health problems and a range of healthcare barriers. Therefore, the aim of this study was to evaluate barriers and explore how primary care providers and autistic adults want to improve their primary healthcare. Semi-structured interviews with three autistic adults, two parents of autistic children and six care providers, were performed to evaluate barriers in Dutch healthcare. Next, in a three-round Delphi-study, 21 autistic adults and 20 primary care providers rated barriers in primary healthcare and assessed recommendations based on usefulness and feasibility. In the thematically analysed interviews, 20 barriers in Dutch healthcare for autistic people were identified. In the Delphi-study, the primary care providers rated the negative impact of most barriers lower than the autistic adults. The Delphi-study resulted in 22 recommendations to improve primary healthcare for autistic adults, focused on: primary care providers (i.e. education in collaboration with autistic people), autistic adults (i.e. improvement of preparation for general practitioner-appointments) and organization of general practice (i.e. enhancement of continuity in care). In conclusion, primary care providers seem to assess healthcare barriers as less impactful than autistic adults. With the use of the Delphi-method, useful and feasible recommendations to improve primary healthcare for autistic adults were identified, based on the needs of autistic adults and primary care providers.

**Lay abstract:**

Autistic adults often encounter different types of healthcare barriers. Because autistic adults also have an increased risk for health problems, the aim of this study was to evaluate barriers and to explore how primary care providers and autistic adults want to improve their primary healthcare. In this co-created study, semi-structured interviews with three autistic adults, two parents of autistic children and six care providers were performed to evaluate barriers in Dutch healthcare. Next, in the survey-study (using the Delphi-method including controlled feedback in three consecutive questionnaires), 21 autistic adults and 20 primary care providers rated the impact of barriers and the usefulness and feasibility of recommendations to improve primary healthcare. In the interviews, 20 barriers in Dutch healthcare for autistic people were found. In the survey-study, the primary care providers rated the negative impact of most barriers lower than the autistic adults. This survey-study resulted in 22 recommendations to improve primary healthcare focused on: primary care providers (including education in collaboration with autistic people), autistic adults (including improvement of preparation for general practitioner-appointments) and organization of general practice (including improvement of continuity in care). In conclusion, primary care providers seem to view healthcare barriers as less impactful than autistic adults. In this co-created study, recommendations to improve primary healthcare for autistic adults were identified, based on the needs of autistic adults and primary care providers. These recommendations provide a basis for primary care providers, autistic adults and their support network to start conversations about, for example, strategies to improve primary care providers’ knowledge, autistic adults’ preparation for a general practitioner-appointment and organization of primary care.

## Introduction

Early mortality alongside an increased prevalence of somatic and psychiatric conditions in autistic adults are pressing problems that ask for urgent improvement of healthcare for autistic adults (Croen et al., 2015; Hand et al., 2020; Hirvikoski et al., 2016; Hwang et al., 2019; Schendel et al., 2016). These health inequities are associated with disparities in access to healthcare, which can result in delayed and insufficient care, higher rates of hospitalization, increased financial costs and premature mortality (Bowles et al., 2002; Lindly et al., 2019; Long et al., 2002; Vecchio et al., 2018). Impaired access to healthcare can be caused by different barriers (Mason et al., 2019). Reducing these barriers in primary healthcare for autistic adults is key, since providing (access to) appropriate healthcare is a main task of general practitioners (GPs) (Mazurek et al., 2020). Despite this need to improve autistic adults’ primary healthcare, there is a lack of recommendations about how to reduce barriers in general practice, from the perspectives of both primary care providers (PCPs) and autistic adults (Gilmore et al., 2022; Walsh et al., 2021).

To understand what type of recommendations could improve primary healthcare, it is crucial to have an impression of barriers that PCPs and autistic adults face in general practice (Doherty et al., 2020; Mason et al., 2019; Walsh, Lydon, O’Dowd, & O’Connor, 2020). Hence, the ‘Barriers to Healthcare Checklist’, an instrument to assess barriers for autistic people in different types of healthcare, and a comparable caregiver-report tool have already been developed (Raymaker et al., 2017; Walsh, Lydon, Hehir, & O’Connor, 2020). Overall, barriers can be divided into four categories: being related to (1) the autistic person, (2) the PCP, (3) the healthcare system or (4) the social environment (Nicolaidis et al., 2015; Walsh, Lydon, O’Dowd, & O’Connor, 2020).

In previous research, various strategies have been suggested to improve primary healthcare for autistic people, such as adjusting lighting in an exam room, minimizing time in the waiting room, providing the PCP with a list of a patient’s needs and education of PCPs (Stein Duker et al., 2019; H. Taylor et al., 2022; Warfield et al., 2015). Furthermore, to improve autistic people’s communication about their healthcare needs, for example, the Academic Autism Spectrum Partnership in Research and Education (AASPIRE) Healthcare toolkit (Nicolaidis et al., 2016) and different versions of an ‘Autism passport’ have been developed (e.g. from Autism Anglia, the British National Autistic Society and the Dutch *Spectrumvisie* (Agterberg & Spectrumvisie, 2018)). In a recent review about interventions improving healthcare access or experiences for autistic people, it was reported that the majority of interventions focused on autistic people and mostly consisted of skills training. Another large part of interventions was provider-focused, mainly comprising education. Organization-focused interventions were less frequently investigated (Walsh et al., 2021). Thus, recommendations to improve primary healthcare for autistic adults should focus on all these three domains: PCPs, autistic adults and the healthcare organization.

It should be noted that the knowledge about barriers and recommendations to improve primary care for autistic adults is based on studies performed in the United States and the United Kingdom (Doherty et al., 2020; Mason et al., 2019; Walsh, Lydon, O’Dowd, & O’Connor, 2020). This limits the generalizability of these barriers and recommendations for improvement, as pathways of funding through insurances and availability of resources can vary between countries and healthcare systems (Faber et al., 2012; Ridic et al., 2012). Moreover, in the Netherlands, the GP is a gatekeeper for referrals from primary care to specialized secondary care (Faber et al., 2012). A team of PCPs in a Dutch GP-office mainly consists of a GP, a general practice nurse (GPN) focused on somatic care and/or a primary care mental health worker (PCMHW, in Dutch: ‘POH-GGZ’). In the Netherlands, these PCMHWs most often have a formal education in psychology (university level) or in nursing focused on psychiatric care (based on vocational education). However, the composition of the types of PCPs employed in a GP-office and the educational levels of these different PCPs also vary between countries (Groenewegen et al., 2015). Therefore, it is needed to specifically explore what type of barriers and recommendations for improvement are relevant in primary care for autistic adults in the Netherlands.

All in all, considering the increased risk of co-occurring conditions and mortality for autistic adults, and the healthcare barriers they experience, improving primary healthcare for autistic adults is a necessity (Raymaker et al., 2017; Walsh et al., 2021). However, recommendations to reduce these barriers and improve primary healthcare for autistic adults specifically in the Netherlands, based on both the needs of Dutch PCPs and autistic adults themselves, are limited, considering the differences in previously studied healthcare systems. Therefore, we identified barriers in Dutch primary care for autistic adults. Subsequently, our main objective was to explore how Dutch PCPs and autistic adults want to improve their primary healthcare; what recommendations do they agree on regarding usefulness and feasibility.

## Methods

### Study design

#### Autistic community involvement

This study was initiated by a project-team of the Dutch Academic Workplace Autism (Academische Werkplaats Autisme), which is a collaborative effort of organizations of autistic people, clinicians and academic institutions, aiming to improve the lives of autistic people based on co-created academic research. The project-team for this study consisted of three healthcare providers, four researchers and three members with lived experience (i.e. two autistic adults and a parent of an autistic child). These project-team members shared their insights into current healthcare barriers and suggestions for reduction of these barriers, which contributed to the development of the research questions and formulation of the Delphi-survey questions.

#### Study design

**Figure 1.**
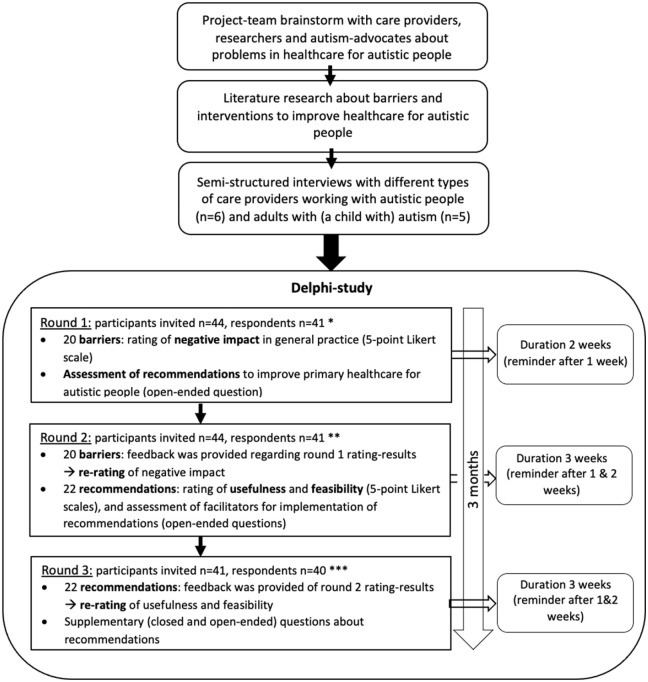
Study design. *21 autistic adults and 20 PCPs; **the same respondents as in round 1; ***20 autistic adults and 20 PCPs.

The total study design is summarized in Figure 1. After a project-team brainstorm and an orientating literature review, 11 semi-structured interviews were performed with primary, secondary and tertiary care providers and (parents of) autistic people. Based on the outcomes of these interviews and the expertise of the project-team members, it was determined that the consecutive Delphi-study should focus on primary healthcare. Since PCPs and autistic approach the investigated barriers and recommendations from other perspectives (being a care provider vs a care receiver), the Delphi-method was used to increase consensus between these two Delphi-panels regarding the usefulness and feasibility of barriers and recommendations. The Delphi-method is specifically suitable for this purpose of creating credible evidence-based recommendations for a healthcare setting, since the Delphi-method results in a smaller range of responses and in more expert consensus (E. Taylor, 2020). This study protocol was approved by the institutional review board of the Leiden University Medical Center (reference no. N21.043).

### Participants

#### Interviews

For the semi-structured interviews, two groups were recruited: (1) people who receive care (*n* = 5) and (2) care providers (*n* = 6). Adults, with a minimum age of 18 years old, with a self-reported autism spectrum disorder (ASD) diagnosis or with an autistic child were included in the first group. In the second group, care providers working with autistic patients in primary, secondary or tertiary care were eligible for inclusion. Purposive sampling was used to include a diverse sample of participants (Moser & Korstjens, 2018). Diversity was evaluated based on age, gender, educational attainment and type of care provider.

#### Delphi-study

The Delphi-panels also consisted of two groups: (1) autistic adults and (2) PCPs. We aimed to include approximately 20 participants in each group, taking possible drop-out into account (Niederberger & Spranger, 2020; E. Taylor, 2020). Criteria for inclusion in the first group were a minimum age of 18 years old, the self-reported presence of an ASD-diagnosis, the ability to independently fill in digital questionnaires and the ability to answer the questionnaires with a broader perspective on the autistic population (e.g. based on experience as an autism-advocate or peer-support worker). In the second group, GPs, GPNs and PCMHWs, with some experience with autistic people in their work in primary care, were eligible for inclusion. Participants were recruited via the network of the project-team members. Purposive sampling was used to include diverse panels (based on age and gender).

### Data collection and analysis

#### Interviews

The list of interview-topics was developed based on literature reviews (Mason et al., 2019; Morris et al., 2019; Walsh, Lydon, O’Dowd, & O’Connor, 2020) and input of the project-team members collected in the preparatory brainstorm-session. With signed informed consent of the participants, the interviews were audio-recorded and transcribed verbatim by the main researcher (E.B.W.).

Transcripts of the interview audio-recordings were uploaded to the software-program ATLAS.ti (ATLAS.ti Scientific Software Development GmbH, version 9). A thematic analysis was performed with a predetermined code-tree (Mason et al., 2019; Morris et al., 2019; Walsh, Lydon, O’Dowd, & O’Connor, 2020). If other relevant themes were encountered, these were openly coded. Central themes were summarized in three main categories: barriers related to PCPs, to autistic people (and their support system) and to healthcare organization.

#### Delphi-study

The Delphi-study was performed with digital questionnaires using Castor EDC software (Castor EDC, 2019). The content and formulation of questions in the surveys were developed in cooperation with the autistic project-team members. An outline of the main questions in each of the three Delphi-rounds is displayed in Figure 1. The list of 20 barriers, first assessed in round 1, was created based on the interview-outcomes, input from the autistic project-team members and literature reviews (Mason et al., 2019; Morris et al., 2019; Walsh, Lydon, O’Dowd, & O’Connor, 2020). In round 2, participants re-rated the barriers that did not reach consensus in round 1. The set of recommendations (first assessed in round 2) was generated out of the participants’ input in open-ended questions in round 1 and the expertise from project-team members. In round 3, recommendations were also re-rated. In all re-rating questions, the participants were provided with feedback about results from the previous round: participant’s own answers and group-results. Based on the outcomes of round 2, additional closed and open-ended questions were incorporated in round 3, aiming for a more detailed understanding of the rating of recommendations.

Consensus in a category (namely, negative impact or priority) of a barrier was reached if ⩾70% of all participants placed it in one of the three main answer options (very low/low, medium or high/very high). Open-ended questions from all three rounds were thematically also analysed in ATLAS.ti, with categorization in themes based on the recommendations and their usefulness and feasibility.

## Results

### Participants

#### Interviews

The five interviewed individuals in the autism-group (two autistic men, one autistic woman and two mothers of an autistic child) were 28–56 years old. One of the autistic men also had a learning disability. The six interviewed care providers (four men and two women) were a psychiatrist, GP, psychotherapist, physician specialized in care for people with a learning disability, PCMHW and physician from a rehabilitation centre.

#### Delphi-study

In total, 21 autistic adults and 20 PCPs participated in the Delphi-study (see Table 1). Most PCPs were female (*n* = 18) and middle-aged (15 PCPs were 41–64 years old). The group of autistic participants consisted of more men (*n* = 8 vs *n* = 2) and were younger than the group of PCPs.

**Table 1.** Delphi-study participants’ characteristics: autistic adults and primary care providers (PCPs).

	Autistic adults (*n* = 21)		PCPs (*n* = 20)		Total group (*N* = 41)	
Gender	*n*	(%)	*n*	(%)	*n*	(%)
Male	8	(38)	2	(10)	10	(24)
Female	13	(62)	18	(90)	31	(76)
Other	0	(0)	0	(0)	0	(0)
Age						
20–30 years	0	(0)	1	(5)	1	(2)
31–40 years	10	(48)	3	(15)	13	(32)
41–50 years	4	(19)	8	(40)	12	(29)
51–64 years	4	(19)	7	(35)	11	(27)
65+ years	3	(14)	1	(5)	4	(10)
Country of birth						
The Netherlands	20	(95)	20	(100)	40	(98)
Outside the Netherlands	1	(5)	0	(0)	1	(2)
Parents’ country of birth						
The Netherlands	20	(95)	20	(100)	40	(98)
Outside the Netherlands	1	(5)	0	(0)	1	(2)
GP/GPN/PCMHW/autistic peer-support worker						
General practitioner (GP)	0	(0)	9	(45)	9	(22)
General practice nurse (GPN)	0	(0)	3	(15)	3	(7)
Primary care mental health worker (PCMHW)	0	(0)	8	(40)	8	(20)
Peer-support worker or autism advocate	21	(100)	0	(0)	21	(51)
Duration of career as a GP						
1–5 years	–	–	1	(5)	–	–
6–10 years	–	–	3	(15)	–	–
11–20 years	–	–	3	(15)	–	–
21+ years	–	–	2	(10)	–	–
Not applicable, because I am not a GP	–	–	11	(55)	–	–
Type of nurses working in your own GP office						
No GPN or PCMHW	–	–	0	(0)	–	–
Only GPN(s)	–	–	0	(0)	–	–
Only PCMHW(s)	–	–	1	(5)	–	–
GPN(s) and PCMHW(s)	–	–	18	(90)	–	–
Not applicable, because I retired during this study	–	–	1	(5)	–	–

### Interviews

As a result of the interviews, 27 barriers in healthcare for autistic people were identified and categorized into three main themes, barriers related to: care providers, autistic people (and their support network) and healthcare organization. These 27 barriers were reduced (with consent of the project-team) to 20 barriers, which were assessed in the Delphi-study (Table 2). This reduction was executed by combining some of the more detailed barriers into broader categories.

**Table 2.** Barriers: negative impact according to autistic adults, primary care providers (PCPs) and the total group of Delphi-participants.^a^

Barriers	Negative impact^b^								
	Low/very low			Medium			High/very high		
	Autistic adults	PCPs	Total group	Autistic adults	PCPs	Total group	Autistic adults	PCPs	Total group
Related to PCPs									
Knowledge about autism	0%	0%	0%	16%	25%	21%	84%	70%	**77%**
Experience with autism	0%	5%	3%	21%	35%	28%	79%	55%	67%
Stigmatizing views about autism	11%	15%	13%	11%	60%	36%	78%	10%	44%
Skills/awareness to individualize care	0%	15%	8%	5%	15%	10%	90%	65%	**78%**
Personal interest in autism	11%	15%	13%	63%	55%	59%	26%	25%	26%
Related to autistic adults									
Knowledge about physical complaints	26%	5%	15%	47%	55%	51%	16%	30%	23%
Rigidity/ability to adjust	5%	5%	5%	21%	20%	21%	68%	75%	**72%**
Sensory regulation of stimuli	5%	5%	5%	21%	45%	33%	74%	50%	62%
Ability to cope with stress/emotions	5%	5%	5%	11%	40%	26%	84%	55%	69%
Recognizing physical complaints	5%	0%	3%	0%	15%	8%	85%	65%	**75%**
Processing of information	5%	5%	5%	10%	25%	18%	75%	65%	**70%**
Communication skills	0%	5%	3%	10%	30%	21%	90%	65%	**77%**
Executive and coordination skills	0%	0%	0%	26%	30%	28%	74%	65%	69%
Feeling of being misunderstood	5%	0%	3%	21%	25%	23%	74%	70%	**72%**
Behavioural problems	26%	5%	15%	26%	50%	39%	42%	40%	41%
Adequate support system	0%	10%	5%	37%	20%	28%	58%	65%	62%
Related to organization of general practice									
Time during appointment with GP	5%	0%	3%	11%	30%	21%	84%	70%	**77%**
Ability to refer to autism-aid	5%	0%	3%	37%	50%	44%	53%	40%	46%
Continuity of care from PCPs	0%	5%	3%	10%	15%	13%	75%	80%	**78%**
Collaboration with other care providers	0%	5%	3%	21%	30%	26%	63%	60%	62%

GP: general practitioner.

a Results from round 1 if consensus was reached in round 1, and results from round 2 if consensus was not reached in round 1. Percentages in bold highlight the barriers about which consensus (⩾70%) was reached in total group of Delphi-participants (C).

b Participants could also answer ‘I do not know/recognize this barrier’. Therefore, the percentages shown of the three categories (none/almost none, a little bit, much/very much) per group (A, B and C) in this table do not always add up to 100%.

### Delphi-study: barriers

The total group of Delphi-participants reached consensus about the (very) high negative impact of nine of the 20 barriers: highlighted in bold in Table 2. None of the other 11 barriers were rated as having a (very) low negative impact. Overall, compared with the autistic participants, PCPs rated the negative impact of most barriers relatively lower. The most prominent difference between the two panels was that only 10% of PCPs rated the negative impact of stigmatizing views of PCPs as (very) high compared with 78% of autistic participants.

### Delphi-study: recommendations

**Figure 2.**
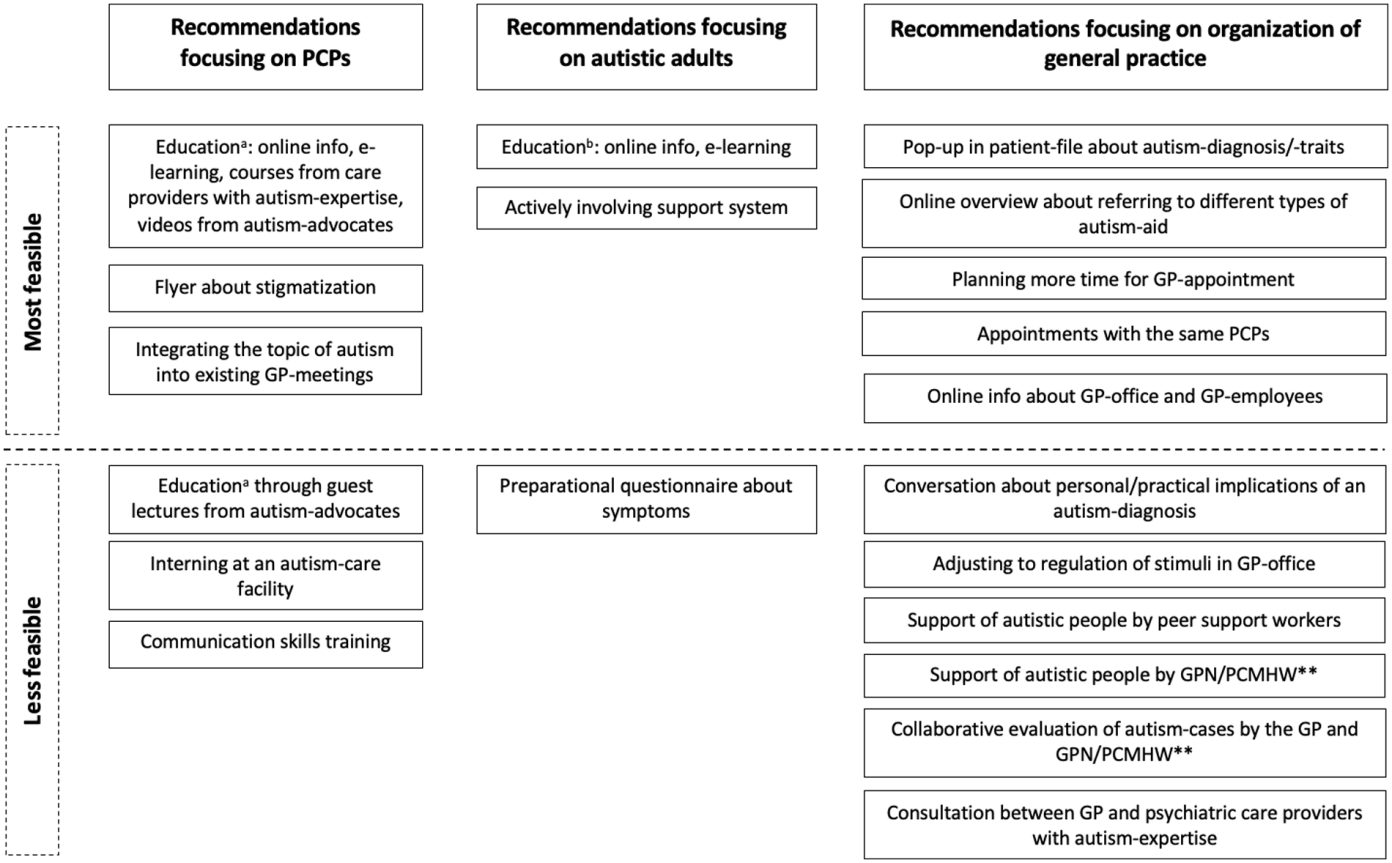
Summary of recommendations to improve primary healthcare, based on both the needs of autistic adults and primary care providers (PCPs)*. ^a^Education about autism, autism-related health problems and autism-related somatic symptoms. ^b^Education about autism-related health problems and physical complaints. *In the total group of Delphi-participants, almost all recommendations (except for a flyer about stigmatization) scored relatively high on usefulness. Therefore, this figure only shows a differentiation in feasibility; **GPN = general practice nurse; PCMHW = primary care mental health worker.

A set of 22 recommendations (1–22) to improve primary healthcare for autistic adults was formulated; for the results of the total group of Delphi-participants, see Table 3 (in which recommendations were numbered (1–22) and highlighted in bold if consensus was reached), and for the results of the subgroups of autistic participants and PCPs, see Table 3b in Supplemental Appendix 1. Overall, the majority of the total group of Delphi-participants rated all recommendations as relatively useful, except for a flyer about stigmatization (7). The feasibility of the total set of recommendations was divided into two main groups: most feasible (1–3,5,7,9,11,12,14–17) and less feasible (4,6,8,10,13,18–22) (see Figure 2), based on the results of the total group of Delphi participants. In the section below, we take a closer look into the recommendations based on a thematic analysis of the Delphi-questionnaires.

**Table 3.** Recommendations: usefulness and feasibility, according to the total group of Delphi-participants.^a^

Recommendations	Usefulness			Feasibility		
	Low /very low	Medium	High/very high	Low/very low	Medium	High/very high
A. Focusing on primary care providers (PCPs) Education . . .						
1. . . . with online info or e-learning	3%	34%	63%	5%	21%	**74%**
2. . . . provided by care providers with autism-expertise	3%	10%	**87%**	0%	46%	54%
3. . . . with videos of autism-advocates	3%	22%	**75%**	5%	25%	**70%**
4. . . . with guest lectures of autism-advocates	6%	17%	**77%**	15%	38%	48%
5. . . . by integrating the topic of autism into existing meetings in the GP-office	5%	25%	**70%**	5%	26%	68%
6. Interning at an autism-care facility	13%	23%	65%	**71%**	19%	0%
7. Flyer about stigmatization	23%	48%	30%	0%	31%	69%
8. Communication training	6%	24%	**71%**	13%	**70%**	18%
B. Focusing on autistic adults						
9. Education with e-health	3%	40%	58%	3%	25%	**73%**
10. Preparational questionnaire	0%	20%	**80%**	5%	31%	64%
11. Actively involving support system	8%	14%	**78%**	0%	33%	68%
C. Focusing on organization of general practice						
12. Pop-up in patient-file about autism-diagnosis/-traits	18%	28%	54%	8%	26%	67%
13. Conversation about personal/practical implications of an autism-diagnosis	15%	13%	**72%**	33%	35%	33%
14. Online overview of autism-aid	6%	24%	**71%**	3%	9%	**88%**
15. Online info about GP-office/-PCPs	3%	23%	**75%**	8%	18%	**75%**
16. Planning more time for GP-appointment	0%	18%	**82%**	5%	28%	68%
17. Appointments with the same PCPs	0%	3%	**97%**	11%	17%	**72%**
18. Adjusting to regulation of stimuli	5%	38%	58%	21%	50%	29%
19. Support of autistic adults by peer-support workers	3%	25%	**73%**	43%	40%	18%
20. Support of autistic adults by GPN/PCMHW^b^	5%	30%	65%	20%	58%	23%
21. Collaborative evaluation of autism-cases by GP and GPN/PCMHW	3%	20%	**78%**	18%	33%	50%
22. Consultation between GP and psychiatric care providers with autism-expertise	8%	22%	**70%**	39%	31%	31%

GP: general practitioner.

a Results from round 2 if consensus was reached in round 2, and results from round 3 if consensus was not reached in round 2. Percentages were calculated based on the number of participants who filled in the answer option (N.B. low/very low, medium or high/very high) divided by the total number of participants who answered the specific question. Percentages in bold highlight the recommendations about which consensus (⩾70%) was reached in the total group of Delphi-participants. The corresponding results of the subgroups of autistic adults and primary care providers can be found in Table 3b in Supplemental Appendix 1.

b GPN = general practice nurse; PCMHW = primary care mental health worker.

#### A. Recommendations focusing on PCPs

Education of PCPs (through online information on a website (1), e-learning (1), with a training of a care provider with autism-expertise (2), through videos (3) or guest lectures (4) of autism-advocates) was assessed as very useful by the majority of the Delphi-participants. Another recommendation entailed integration of this education into existing meetings in the GP-office (5). Autistic participants often commented that education will be more effective if a diverse team of autistic people with lived experience is involved in the development of educational programmes. Education could, for example, provide information about ASDs, healthcare barriers autistic people face and health problems and somatic symptoms related to autism. PCPs mentioned that the feasibility of educational interventions will be higher if accreditation is added, which makes it more appealing to partake in a training or e-learning.

The feasibility of a communication skills training (8) was evaluated less highly than most other recommendations. Participants mentioned the complexity of such a training and doubted PCPs’ motivation to voluntarily participate. The idea of letting a PCP be an intern (for a day) at an autism-care facility (6), to increase experience with autistic people, was assessed as least feasible because of a lack of time. In both groups, but particularly in autistic participants, a flyer about stigmatization (7) was the only recommendation that was rated with relatively low usefulness. Autistic participants mainly doubted if this flyer could really change PCPs’ behaviour towards autistic adults.

#### B. Recommendations focusing on autistic adults

In this category, active involvement of the autistic person’s support system (11) was assessed with (very) high usefulness and relatively high feasibility. Next, education for autistic adults, using e-health about recognizing (psycho)somatic complaints (9), was not considered as most useful, but the feasibility was evaluated as (very) high. Autistic participants doubted if education with e-health could actually improve body awareness and recognition of physical complaints, because the ability to apply generalized information to yourself can be impaired in autistic people. Finally, a preparational questionnaire about physical complaints to fill in before the GP-appointment (10) was assessed as (very) useful by both groups. However, this preparational questionnaire was seen as less feasible, mainly by PCPs, because of the expected difficulty to develop a questionnaire covering all types of physical complaints.

#### C. Recommendations focusing on the organization of general practice

Both groups considered planning appointments with the same PCPs (17) as the most useful recommendation (across all categories), because predictability and consistency in care are very important for autistic adults. Moreover, the feasibility of this recommendation was assessed as (very) high. The recommendation concerning an online overview of different types of autism-aid (14) was also attributed with (very) high usefulness and feasibility by the total group of Delphi-participants. Specifically, to provide individualized care, PCPs need to know to who and how to refer a patient, based on regional information about available services. The recommendation regarding the availability of online information (15) (such as pictures on the website of the GP-office about the employees, waiting room and doctor’s office) was rated as (very) useful and (very) feasible, according to the total group of Delphi-participants. PCPs did comment that maintaining an up-to-date website about employees could be challenging, because of the often rapidly changing composition of teams in a GP-office. Planning more time per GP-appointment (16) was also assessed with (very) high usefulness and relatively high feasibility. This recommendation could be useful if an autistic person, for example, needs more time to process information. Its feasibility depends on the ability to claim a longer consultation through healthcare insurance.

The recommendations in category C that the majority of Delphi-participants perceived as (very) useful, but were assessed with relatively lower feasibility, were: conversation with a PCP about personal/practical implications of an autism-diagnosis (13), regulate/adjust stimuli in the GP-office (18), support of autistic adults by peer-support workers (19) or by GPNs/PCMHWs (20), collaborative evaluations of autism-cases by the PCPs working in the GP-office (21) and consultation between the GP and psychiatric care providers with autism-expertise (22). Suggested facilitators to improve the feasibility of each recommendation, respectively, included planning a separate appointment with a PCP about personal healthcare barriers (13), considering to plan appointments outside rush-hours at the GP-office to reduce an overload of stimuli (18), informing PCPs about autistic peer-support workers and where to find them (19), educating GPNs/PCMHWs about autism (20), adding these autism-case-evaluations to existing complex-case evaluations (21) and expanding consultation to regional autism-networks, instead of only consulting psychiatric facilities (22).

Finally, the recommendation with the relatively lowest usefulness in this category was the use of a pop-up in the digital patient-file about the presence of an autism-diagnosis and/or autistic traits (12). Autistic Delphi-participants commented that this pop-up could be stigmatizing and that its usefulness depends on PCPs’ knowledge about autism. However, PCPs mentioned that this type of pop-ups is often closed before the content is read, because there are already too many pop-ups in patient-files.

## Discussion

The need to improve healthcare for autistic adults is evident because of the multiple barriers autistic adults and their PCPs often face in accessing and providing healthcare. In this study, we explored how PCPs and autistic adults want to improve their primary healthcare. First, 20 barriers in Dutch primary healthcare were investigated; all these 20 barriers were assessed with a medium to (very) high negative impact in general practice by the majority of Delphi-participants. However, PCPs rated the negative impact of most barriers relatively lower than the autistic participants. This discrepancy emphasizes the need to better recognize and decrease these barriers in primary care, as PCPs might not always be able to adequately assess the impact of barriers in primary care for autistic adults. Next, we conceptualized recommendations to improve primary healthcare for autistic adults, based on the perspectives of autistic adults and PCPs. All recommendations (except for the flyer about stigmatization) were assessed as relatively useful. Since the feasibility results were more divergent, all recommendations were divided into two categories: most feasible and relatively less feasible for implementation.

The identified recommendations regarding PCPs mainly involved education. In the previous literature, mostly online educational interventions to increase PCPs’ knowledge about autism have been suggested as well (Nicolaidis et al., 2015; Walsh et al., 2021). The results from this study add that it should be considered to complement educational courses with input from a diverse group of autistic people with lived experience, so they can illustrate the healthcare barriers they face. A facilitator for educational interventions is the inclusion of accreditation, since this could make it more appealing for PCPs to partake in a course or an e-learning.

An informational flyer about stigmatization was assessed as less useful, partly because of the uncertainty in autistic participants about if this flyer could actually result in changes of PCPs’ behaviour/communication towards autistic adults. However, the need for interventions to reduce stigma (Mason et al., 2019; Nicolaidis et al., 2015) was emphasized by the striking discrepancy we found between autistic participants’ and primary PCPs’ perceptions of the negative impact of the barrier involving PCPs’ stigmatizing views. Moreover, this disagreement between the autistic participants and PCPs regarding the negative impact of stigmas asks for more research into the presence and different types of stigmatic beliefs among PCPs and into the possible negative and positive impact of these stigmas on primary care for autistic adults.

Previous research regarding interventions focusing on autistic people mostly included behavioural interventions (Walsh et al., 2021). However, in this study, the recommendations focusing on autistic adults were directed at enhancing their own knowledge about how their body works, to more actively involve their support system and to improve their preparation for a GP-appointment. The latter recommendation is supported by the British online AASPIRE Healthcare toolkit (Nicolaidis et al., 2016; toolkit available at: http://autismandhealth.org) and a recently developed pre-appointment health check (H. Taylor et al., 2022), since this toolkit and health check are partly aimed to improve an autistic person’s preparation for a (primary) healthcare appointment.

With regard to the third category of recommendations focusing on organizational aspects of general practice, previous studies about general healthcare or primary care reported some comparable recommendations. These are, for example, the availability of a list of local supportive services, enhancement of the interior of the GP-office (e.g. by adjusting the light in the waiting room and reducing waiting times), consistency in care, clarification of the autistic person’s needs and personal implications of the autism-diagnosis and improvement of collaboration between different care providers (H. Taylor et al., 2022; Walsh et al., 2021; Warfield et al., 2015). Thus, this study supports these recommendations, which would therefore be useful and feasible for implementation in both the Netherlands as in other countries. In this category of recommendations focusing on organizational aspects, this study adds some recommendations to previous literature regarding the implementation of more support by primary practice nurses (GPN/PCMHW) and by autistic peer-support workers. It should be noted that the implementation of support by practice nurses could vary between countries, as the level/type of education and responsibilities/tasks of practice nurses are different in each healthcare system. Finally, while the concept of peer support by autistic individuals is not new, future implementation of autistic peer-support workers in primary care also depends on the possibilities of developments in the healthcare system in a country, since this asks for training programmes, reforms in primary care and financial support (Shea et al., 2022).

### Strengths and limitations

The main strength of this study is the inclusion of a large Delphi-panel consisting of 20 PCPs and 21 autistic adults, in order to establish recommendations based on the perspectives and needs of both stakeholders. Also, the drop-out in our Delphi-study was very low, which magnifies the validity of the Delphi-method. Moreover, this study design was co-created with autistic project-team members with lived experience, which increases the relevance of this study (Fletcher-Watson et al., 2019). Furthermore, a thorough step-by-step study design (including qualitative methods), enforced by regular project-team feedback-rounds, was executed.

We aimed to include diverse panels of Delphi-participants, but we experienced difficulties to recruit participants with different ethnic and cultural backgrounds. This limits this study in the extent that the results cannot be generalized to autistic adults of all types of background, while this is important in the fight against racism in autism research and practice (Jones et al., 2020). Also, this study results can only be related to the primary care for autistic adults, as we purposively only included adults in the Delphi-study. This adult study population was chosen because we hypothesized that barriers and recommendations for improvement of primary care could be very different for autistic children and their parents. The latter would ask for separate research into recommendations to improve Dutch primary care for autistic children. It should also be noted that the Delphi-participants had to independently complete the questionnaires. Therefore, this study results cannot be generalized to autistic adults with higher levels of support needs. Another limitation of this study design was the lack of more detailed information regarding the participating PCPs’ familiarity with autistic people, while it was an inclusion criterium to have some experience with autistic patients. In future research, it could be attempted to include autistic PCPs, because it would be interesting to investigate if autistic PCPs, for example, experience less or other barriers in the care for autistic adults. Furthermore, we explored how PCPs and autistic adults want to improve their primary care, but we did not investigate the practical implementation of our recommendations. Thus, from this study, no conclusions can be drawn regarding the effectiveness of implementation in general practice.

### Implications

Most barriers we investigated were assessed with relatively lower negative impact on primary healthcare for autistic adults by PCPs than by autistic participants in our Delphi-study. It could be hypothesized that a better understanding among PCPs regarding the possible impact of barriers in primary care for autistic adults might contribute to both earlier recognition and more effective implementation of recommendations to improve primary care. Thus, this first finding suggests that education for PCPs about the impact of these barriers could be relevant. The recommendations assessed in this study provide a basis for PCPs, autistic adults and their support/social network to start conversations about their needs in providing, accessing and improving healthcare; recommendations in different categories can guide future implementation in general practice. A next step for future studies is to investigate the effectiveness of our recommendations implemented in general practice. Furthermore, several recommendations investigated in this study point in the direction of the use of both autism-advocates (autistic individuals with lived experience) to improve education for PCPs and autistic peer-support workers to help other autistic adults navigate through primary care. However, since these recommendations were assessed as relatively less feasible, the implementation of these recommendations involving autistic peer-support workers should be investigated in more depth. Finally, this study only focused on improvement of primary care for autistic adults, but the recommendations we investigated might also be applicable for improvement of care for other types of adults experiencing comparable barriers in primary care. For example, the individualization of care based on personal traits or barriers, the implementations of peer-support workers, the active involvement of the social network or the enhancement of collaboration between different types of care providers could all possibly be recommendations that might be helpful for improvement of primary care for adults without autism experiencing comparable barriers.

## Conclusion

Autistic adults have an increased risk for co-occurring conditions and for mortality, while also facing impaired access to healthcare due to different types of barriers. Surprisingly, PCPs seem to view most healthcare barriers we investigated as less impactful than autistic adults. All in all, this Delphi-study resulted in 22 recommendations to improve primary healthcare for autistic adults, based on the perspectives of both autistic adults and PCPs, and related to PCPs, autistic adults and the organization of general practice. For example, these recommendations can be used as guidance to increase PCPs’ knowledge, enhance autistic adults’ preparation for a GP-appointment, personalize healthcare for adults with autism and improve organizational aspects of primary healthcare for autistic adults. Future research into the outcomes of implementation of our recommendations in general practice is needed.

## Supplemental Material

sj-docx-1-aut-10.1177_13623613231172865 – Supplemental material for How do primary care providers and autistic adults want to improve their primary care?: A Delphi-study Click here for additional data file. Supplemental material, sj-docx-1-aut-10.1177_13623613231172865 for How do primary care providers and autistic adults want to improve their primary care?: A Delphi-study by Eva B Warreman, Wietske A Ester, Hilde M Geurts, Robert RJM Vermeiren and Laura A Nooteboom in Autism
