# Supplementary material for: How do primary care providers and autistic adults want to improve their primary care? A Delphi-study
Source: Autism. 2023 May 16;28(2):449–60. doi: 10.1177/13623613231172865 (PMC10851648; doi:10.1177/13623613231172865)
Supplement: sj-docx-1-aut-10.1177_13623613231172865 – Supplemental material for How do primary care providers and autistic adults want to improve their primary care?: A Delphi-study [file sj-docx-1-aut-10.1177_13623613231172865.docx]

**Appendix 1**

Table 3b. Recommendations: usefulness and feasibility, according to the subgroups of autistic adults (AA) and primary care providers (PCPs)

| **Recommendations** | **Usefulness** | | | | | | **Feasibility** | | | | | |
| --- | --- | --- | --- | --- | --- | --- | --- | --- | --- | --- | --- | --- |
|  | *Low /*  *very low* | | *Medium* | | *High /*  *very high* | | *Low /*  *very low* | | *Medium* | | *High / very high* | |
|  | AA | PCPs | AA | PCPs | AA | PCPs | AA | PCPs | AA | PCPs | AA | PCPs |
| 1. Focusing on primary care providers (PCPs)   Education …  1. … with online info or e-learning  2. … provided by care providers with autism-expertise  3. … with videos of autism-advocates  4. … with guest lectures of autism-advocates  5. … by integrating the topic of autism into existing meetings in the GP-office  6. Interning at an autism-care facility  7. Flyer about stigmatization  8. Communication training | 6%  5% 6%  11%  5%  20%  30%  12% | 0%  0% 0%  0%  5%  5%  15%  0% | 41%  14% 17%  11%  20%  25%  40%  12% | 27%  6% 28%  24%  30%  20%  55%  35% | 53%  81% 78%  78%  75%  55%  30%  77% | 73%  94% 72%  77%  65%  75%  30%  65% | 10%  0% 5%  5%  0%  72%  0%  5% | 0%  0% 5%  25%  10%  85%  0%  20% | 10%  37% 10%  35%  28%  28%  16%  75% | 32%  55% 40%  40%  25%  15%  45%  65% | 80%  63% 85%  60%  72%  0%  84%  20% | 68%  45% 55%  35%  65%  0%  55%  15% |
| 1. Focusing on autistic adults   9. Education with e-health  10. Preparational questionnaire  11. Actively involving support system | 5%  0%  16% | 0%  0%  0% | 40%  20%  11% | 40%  20%  17% | 55%  80%  74% | 60%  80%  83% | 0%  10%  0% | 5%  0%  0% | 25%  20%  25% | 25%  42%  40% | 75%  70%  75% | 70%  58%  60% |
| 1. Focusing on organization of general practice   12. Pop-up in patient-file about autism-diagnosis/-traits  13. Conversation about personal/practical implications of an autism-diagnosis  14. Online overview of autism-aid  15. Online info about GP-office/-PCPs  16. Planning more time for GP-appointment  17. Appointments with the same PCPs  18. Adjusting to regulation of stimuli  19. Support of autistic people by peer support workers  20. Support of autistic people by GPN/PCMHW **  21. Collaborative evaluation of autism-cases by GP and GPN/PCMHW  22. Consultation between GP and psychiatric care providers with autism-expertise | 25% 26%  12%  0%  0% 0%  5%  5%  10%  0%  5% | 11% 5%  0%  5%  0% 0%  5%  0%  0%  5%  11% | 30% 11%  24%  20%  26% 5%  35%  15%  10%  20%  26% | 26% 15%  24%  25%  11% 0%  40%  35%  50%  20%  17% | 45% 63%  65%  80%  74% 95%  60%  80%  80%  80%  68% | 63% 80%  77%  70%  90% 100%  55%  65%  50%  75%  72% | 11% 35%  0%  5%  5% 6%  16%  35%  10%  10%  32% | 5% 30%  6%  10%  5% 17%  26%  50%  30%  25%  45% | 21% 30%  6%  10%  25% 28%  53%  40%  60%  35%  21% | 30% 40%  12%  25%  30% 6%  47%  40%  55%  30%  40% | 68% 35%  94%  85%  70% 67%  32%  25%  30%  55%  47% | 65% 30%  82%  65%  65% 78%  26%  10%  15%  45%  15% |

** Results from round 2 if consensus was reached in round 2, and results from round 3 if consensus was not reached in round 2. Percentages per group (autistic adults: n=21; PCPs: n=20) were calculated based on the number of participants in the respective group that filled in the answer option (N.B. low / very low, medium, or high / very high) divided by the total number of participants that answered the specific question in that group. ** GPN = general practice nurse; PCMHW = primary care mental health worker*
